# Supplementary material for: A Likelihood Approach for Real-Time Calibration of Stochastic Compartmental Epidemic Models
Source: PLoS Comput Biol. 2017 Jan 17;13(1):e1005257. doi: 10.1371/journal.pcbi.1005257 (PMC5240920; doi:10.1371/journal.pcbi.1005257)
Supplement: S1 File — (TAR.GZ) [file pcbi.1005257.s014.tar.gz › HSPH_Online-SI-Revision/Additions/PF09/n100-extreme-SEITR/n100-extreme-SEITR_PF_table-8.pdf]

1	R0	{1.38654, 9.85565}	{1.79987, 19.9962}	{{1.7438, 20.}, {1.85546, 20.}}	{29.8103, 102.891}	inf	369.	842.079	{710.18, 1021.09}	61.6275	0.204157	newly	522.	336.71	{276., 405.}	35.4962	0.195315	Reff	1.36736	1.78029	{1.72749, 1.83439}	30.1984	ar	92 379.	77 708.	{75 219., 79 934.}	15.8814
2	R0	{2.36629, 12.2029}	{2.01833, 16.7321}	{{1.35825, 11.3999}, {2.7777, 20.}}	{23.2375, 41.7153}	inf	10.	238.44	{86.0597, 417.827}	1603.14	1.16193	newly	20.	132.47	{46., 227.}	562.35	0.734993	Reff	2.3656	1.98838	{1.34951, 2.76502}	23.3806	ar	99 022.	74 778.9	{48 431., 93 141.}	24.4825
3	R0	{2.32253, 12.836}	{1.92589, 13.8085}	{{1.19018, 6.16453}, {2.75598, 19.5242}}	{25.1494, 33.2962}	inf	18.	164.358	{8.26673, 388.95}	528.844	0.745222	newly	26.	116.21	{6., 283.}	374.115	0.705922	Reff	2.32139	1.8767	{1.15086, 2.7524}	25.8435	ar	98 997.	64 145.3	{28 247., 91 955.}	35.2048
4	R0	{1.79075, 11.2058}	{1.95998, 15.9684}	{{1.33508, 8.40707}, {2.75432, 20.}}	{26.6709, 50.0935}	inf	68.	241.71	{97.943, 432.941}	151.849	0.341812	newly	88.	135.36	{52., 232.}	72.9545	0.233845	Reff	1.78676	1.93701	{1.33299, 2.74319}	26.1746	ar	96 920.	72 110.1	{45 882., 92 979.}	25.5983
5	R0	{2.26952, 9.62371}	{1.93444, 13.9966}	{{1.19018, 6.18069}, {2.85956, 19.5242}}	{25.329, 57.2062}	inf	2.	161.443	{8.26673, 358.316}	3141.14	1.32457	newly	5.	112.05	{4., 261.}	2157.	1.22322	Reff	2.26934	1.8832	{1.15086, 2.7524}	25.5838	ar	98 881.	63 657.6	{19 155., 92 658.}	35.6221
6	R0	{1.57191, 6.89004}	{2.02587, 15.5899}	{{1.36435, 10.0227}, {2.96567, 20.}}	{35.5934, 126.267}	inf	11.	223.485	{64.7351, 442.751}	1762.38	1.16807	newly	22.	133.61	{34., 252.}	507.864	0.673885	Reff	1.57147	1.99725	{1.36328, 2.94606}	34.2529	ar	95 046.	74 193.5	{48 164., 94 837.}	22.0229
7	R0	{1.99226, 17.2339}	{1.81321, 19.9949}	{{1.72919, 20.}, {1.87221, 20.}}	{8.98732, 16.021}	inf	713.	946.302	{808.456, 1113.19}	12.7451	0.0597545	newly	648.	379.91	{317., 456.}	41.3719	0.236319	Reff	1.94486	1.79044	{1.7125, 1.84392}	7.94001	ar	97 992.	78 042.3	{75 124., 80 459.}	20.3585
8	R0	{2.63369, 15.1879}	{1.99009, 15.9232}	{{1.32006, 8.16704}, {2.75954, 20.}}	{27.5694, 24.1189}	inf	35.	237.433	{94.4452, 399.316}	356.601	0.593276	newly	41.	138.45	{52., 223.}	241.488	0.468291	Reff	2.6309	1.96731	{1.3073, 2.74713}	27.9451	ar	99 451.	73 522.7	{45 163., 93 133.}	26.0714
9	R0	{2.53271, 9.90552}	{1.93444, 13.9966}	{{1.19018, 6.18069}, {2.85956, 19.5242}}	{27.7432, 53.9286}	inf	2.	161.443	{8.26673, 358.316}	7981.17	1.66562	newly	8.	112.05	{4., 261.}	1319.13	1.06492	Reff	2.53258	1.8832	{1.15086, 2.7524}	28.8967	ar	99 355.	63 657.6	{19 155., 92 658.}	35.9292
10	R0	{2.48791, 18.9003}	{1.90958, 17.391}	{{1.50259, 13.3866}, {2.61297, 20.}}	{25.2219, 12.1165}	inf	205.	363.096	{211.554, 600.184}	34.997	0.130611	newly	173.	181.13	{86., 292.}	36.3526	0.158649	Reff	2.47216	1.89005	{1.4953, 2.60036}	25.3954	ar	99 291.	75 307.6	{60 706., 92 880.}	24.1547
11	R0	{2.84414, 18.4765}	{1.86741, 19.2652}	{{1.62675, 15.9743}, {2.12415, 20.}}	{34.342, 8.95357}	inf	68.	403.434	{276.394, 537.86}	291.683	0.578039	newly	76.	173.22	{110., 247.}	127.921	0.336345	Reff	2.83774	1.84587	{1.62227, 2.10123}	34.9529	ar	99 654.	77 898.9	{69 054., 85 838.}	21.8306
12	R0	{2.29369, 14.622}	{1.84443, 17.703}	{{1.51579, 14.0414}, {2.05569, 20.}}	{21.8231, 26.0176}	inf	100.	314.047	{182.747, 470.089}	122.729	0.323099	newly	114.	152.14	{74., 242.}	47.9474	0.167497	Reff	2.28683	1.82103	{1.49725, 2.01814}	22.5352	ar	98 982.	74 533.6	{61 527., 82 693.}	24.6999
13	R0	{2.73413, 16.7529}	{2.06903, 17.4382}	{{1.49021, 12.4991}, {3., 20.}}	{26.5487, 19.3053}	inf	62.	315.105	{183.302, 540.547}	303.981	0.568286	newly	67.	162.14	{78., 267.}	143.91	0.343762	Reff	2.72932	2.04919	{1.4745, 2.98706}	27.0048	ar	99 543.	78 377.1	{59 528., 95 568.}	21.2631
14	R0	{1.79554, 16.5943}	{1.80514, 19.9913}	{{1.75331, 20.}, {1.85115, 20.}}	{1.96534, 20.471}	inf	652.	1117.85	{965.384, 1290.93}	17.8602	0.0695062	newly	541.	447.46	{371., 522.}	17.5786	0.0865286	Reff	1.75512	1.77568	{1.72913, 1.81818}	2.16488	ar	96 821.	78 111.4	{76 341., 79 958.}	19.3239
15	R0	{1.91429, 13.7771}	{1.78013, 20.}	{{1.72084, 20.}, {1.83558, 20.}}	{7.00822, 45.1684}	inf	266.	814.651	{671.812, 981.405}	114.948	0.328544	newly	278.	325.87	{265., 386.}	19.2554	0.0740822	Reff	1.89691	1.76141	{1.70385, 1.81642}	7.14299	ar	97 541.	76 934.3	{74 448., 79 004.}	21.1262
16	R0	{1.42094, 10.5871}	{1.86139, 19.1513}	{{1.62617, 15.5663}, {2.12952, 20.}}	{30.9973, 80.8925}	inf	260.	581.557	{395.478, 815.966}	54.0622	0.171697	newly	379.	252.44	{161., 369.}	34.0739	0.203182	Reff	1.40736	1.83445	{1.61546, 2.08621}	30.3475	ar	93 044.	77 898.	{69 030., 85 992.}	16.2783
17	R0	{1.45515, 6.16466}	{1.93444, 13.9966}	{{1.19018, 6.18069}, {2.85956, 19.5242}}	{41.4101, 129.305}	inf	5.	161.443	{8.26673, 358.316}	5292.1	1.51408	newly	11.	112.05	{4., 261.}	939.	0.961555	Reff	1.45502	1.8832	{1.15086, 2.7524}	39.4953	ar	93 254.	63 657.6	{19 155., 92 658.}	31.7624
18	R0	{2.85669, 13.2416}	{2.01452, 15.7195}	{{1.3547, 10.8872}, {2.96567, 20.}}	{30.6594, 27.5434}	inf	3.	216.118	{64.7351, 442.751}	1864.71	1.20058	newly	8.	126.87	{34., 239.}	1485.88	1.09866	Reff	2.85618	1.98682	{1.34436, 2.9493}	31.3699	ar	99 644.	73 804.4	{47 098., 94 837.}	25.9319
19	R0	{2.56417, 17.9558}	{1.80982, 19.7866}	{{1.69788, 19.533}, {2.0689, 20.}}	{29.4188, 11.1319}	inf	91.	434.429	{311.807, 670.295}	180.277	0.431006	newly	91.	179.33	{120., 299.}	97.0659	0.271864	Reff	2.55604	1.79518	{1.69131, 1.97091}	29.7672	ar	99 389.	76 640.1	{72 767., 84 261.}	22.8888
20	R0	{2.27517, 11.1864}	{2.1189, 15.556}	{{1.38402, 8.1266}, {3., 20.}}	{22.939, 49.3388}	inf	8.	247.69	{71.0994, 430.309}	1448.06	1.09579	newly	17.	175.88	{42., 369.}	934.588	0.8612	Reff	2.27446	2.08511	{1.38322, 2.95768}	22.7282	ar	98 894.	77 418.1	{51 523., 95 288.}	21.716
21	R0	{2.35735, 10.4735}	{1.93361, 13.9067}	{{1.20956, 6.16453}, {2.85956, 19.5242}}	{25.6296, 48.3655}	inf	5.	161.279	{8.26673, 358.316}	3137.85	1.32479	newly	5.	115.68	{4., 271.}	2229.6	1.22787	Reff	2.35707	1.88351	{1.15086, 2.7524}	26.2824	ar	99 036.	63 906.7	{27 943., 92 658.}	35.4712
22	R0	{1.79551, 11.9179}	{1.91761, 17.1973}	{{1.47459, 12.6676}, {2.59858, 20.}}	{18.4736, 47.2251}	inf	143.	398.219	{223.299, 731.254}	81.3487	0.220306	newly	205.	199.72	{110., 340.}	38.0878	0.171694	Reff	1.78616	1.89121	{1.44925, 2.58461}	17.8844	ar	97 033.	75 314.1	{57 614., 92 255.}	22.383
23	R0	{1.23545, 6.31687}	{2.08182, 17.3496}	{{1.49873, 12.4739}, {2.8752, 20.}}	{68.5069, 174.655}	inf	66.	401.902	{185.037, 735.14}	226.75	0.460207	newly	213.	210.25	{98., 358.}	43.6573	0.199338	Reff	1.23161	2.05245	{1.48536, 2.68113}	66.6483	ar	88 739.	78 970.9	{60 661., 94 524.}	14.8363
24	R0	{2.24089, 10.1789}	{2.09862, 15.2648}	{{1.3704, 8.20312}, {2.96542, 20.}}	{23.2428, 57.1195}	inf	4.	212.61	{70.8946, 370.187}	3443.49	1.46842	newly	6.	149.2	{36., 282.}	2386.67	1.25916	Reff	2.24062	2.0674	{1.36923, 2.95323}	22.9412	ar	98 868.	76 566.1	{49 900., 94 795.}	22.5572
25	R0	{1.57298, 10.2607}	{1.8499, 18.6796}	{{1.6178, 15.2378}, {2.11645, 20.}}	{18.3495, 82.1472}	inf	107.	435.632	{248.543, 611.2}	206.783	0.459745	newly	146.	197.9	{97., 284.}	52.3151	0.180666	Reff	1.56721	1.83245	{1.5894, 2.09352}	17.6561	ar	94 957.	77 009.	{68 421., 85 491.}	18.9188
26	R0	{1.2154, 8.2576}	{1.8207, 18.4414}	{{1.58825, 15.1427}, {2.10206, 20.}}	{49.8025, 123.326}	inf	374.	761.644	{471.444, 1155.33}	64.7082	0.1959	newly	559.	339.92	{181., 493.}	39.6208	0.244978	Reff	1.19834	1.78329	{1.57063, 2.0292}	48.8138	ar	88 421.	75 851.2	{66 431., 85 175.}	14.2158
27	R0	{2.22841, 18.7983}	{1.7904, 19.5334}	{{1.57848, 19.1649}, {2.0781, 20.}}	{19.6558, 7.11326}	inf	579.	633.432	{402.645, 998.125}	33.3764	0.180569	newly	487.	261.57	{161., 410.}	46.4209	0.297643	Reff	2.1875	1.76219	{1.56703, 1.96114}	19.4425	ar	98 756.	75 530.2	{66 273., 84 149.}	23.5184
28	R0	{1.27778, 5.36767}	{1.94437, 13.8405}	{{1.19018, 6.16453}, {2.85956, 19.5242}}	{54.6151, 159.969}	inf	15.	167.711	{16.5687, 388.95}	846.668	0.86849	newly	37.	127.67	{6., 270.}	275.27	0.605002	Reff	1.27717	1.89635	{1.15086, 2.7524}	52.1074	ar	89 843.	65 702.7	{28 247., 92 658.}	27.8095
29	R0	{2.38042, 15.8901}	{2.03516, 17.5762}	{{1.51081, 12.5712}, {2.70037, 20.}}	{22.9662, 20.7013}	inf	75.	318.735	{189.666, 523.005}	153.335	0.364016	newly	93.	162.03	{83., 267.}	80.8925	0.2248	Reff	2.37421	2.01636	{1.50913, 2.68394}	23.3471	ar	99 112.	77 846.2	{60 623., 94 064.}	21.4564
30	R0	{2.53188, 12.7607}	{1.93444, 13.9966}	{{1.19018, 6.18069}, {2.85956, 19.5242}}	{27.7319, 33.3867}	inf	1.	161.443	{8.26673, 358.316}	3141.14	1.32457	newly	3.	112.05	{4., 261.}	3649.	1.4032	Reff	2.5317	1.8832	{1.15086, 2.7524}	28.8838	ar	99 317.	63 657.6	{19 155., 92 658.}	35.9047
31	R0	{1.24482, 10.5471}	{1.8719, 19.3978}	{{1.65355, 16.0638}, {2.1117, 20.}}	{50.3748, 83.9159}	inf	904.	1707.42	{1390.35, 2312.12}	34.8428	0.123115	newly	1087.	714.58	{580., 954.}	34.3937	0.189821	Reff	1.19689	1.80427	{1.60498, 1.91982}	50.746	ar	89 097.	79 883.7	{71 679., 85 909.}	10.3408
32	R0	{2.30446, 17.9607}	{1.89823, 17.2209}	{{1.48888, 12.8418}, {2.56735, 20.}}	{22.4614, 13.9326}	inf	288.	392.219	{215.998, 692.805}	37.8458	0.177183	newly	254.	194.9	{89., 338.}	38.4094	0.218727	Reff	2.28427	1.87368	{1.48293, 2.54595}	22.7571	ar	99 028.	74 830.4	{59 691., 92 042.}	24.4351
33	R0	{2.31579, 11.8236}	{1.91401, 13.8175}	{{1.18976, 6.16453}, {2.75598, 19.5242}}	{25.2495, 37.6077}	inf	22.	167.293	{16.5687, 362.69}	516.599	0.742564	newly	33.	117.53	{6., 261.}	283.97	0.642579	Reff	2.31442	1.86453	{1.15086, 2.74816}	25.9941	ar	99 012.	63 462.4	{17 527., 91 044.}	35.9043
34	R0	{1.6678, 10.6744}	{1.91578, 18.1361}	{{1.47298, 14.507}, {2.63716, 20.}}	{20.5318, 70.1564}	inf	174.	379.757	{192.852, 646.569}	66.2648	0.193443	newly	267.	177.04	{82., 279.}	43.1461	0.263254	Reff	1.65861	1.89244	{1.46832, 2.60071}	19.6773	ar	95 809.	75 404.8	{58 221., 92 896.}	21.3055
35	R0	{1.65476, 7.07559}	{1.93361, 13.9067}	{{1.20956, 6.16453}, {2.85956, 19.5242}}	{32.2025, 102.25}	inf	1.	161.279	{8.26673, 358.316}	1929.84	1.15416	newly	10.	115.68	{4., 271.}	1076.6	0.99683	Reff	1.65458	1.88351	{1.15086, 2.7524}	31.0679	ar	95 746.	63 906.7	{27 943., 92 658.}	33.2539
36	R0	{2.71807, 18.5959}	{1.93607, 18.4086}	{{1.56303, 14.8981}, {2.57777, 20.}}	{28.978, 10.7488}	inf	74.	323.473	{162.988, 519.467}	263.453	0.521087	newly	61.	152.33	{64., 254.}	152.803	0.358013	Reff	2.71258	1.90749	{1.55708, 2.56568}	29.8709	ar	99 540.	77 234.5	{64 892., 92 023.}	22.4086
37	R0	{2.6202, 12.7552}	{2.01839, 17.3156}	{{1.50151, 11.7398}, {2.67537, 20.}}	{24.9973, 41.1907}	inf	2.	311.251	{163.92, 497.727}	4346.44	1.60075	newly	7.	159.64	{71., 277.}	2180.57	1.30071	Reff	2.61991	2.00046	{1.48149, 2.65473}	25.4778	ar	99 426.	77 782.5	{60 260., 93 761.}	21.7685
38	R0	{1.47672, 9.81651}	{1.85479, 19.2892}	{{1.62251, 15.5771}, {2.12609, 20.}}	{25.6023, 96.4978}	inf	196.	546.072	{368.859, 792.945}	65.2289	0.199151	newly	329.	233.34	{140., 341.}	31.2219	0.183547	Reff	1.46491	1.82694	{1.61241, 2.05756}	24.714	ar	93 815.	77 676.9	{69 029., 85 500.}	17.202
39	R0	{1.90004, 12.3313}	{1.98048, 17.9155}	{{1.54363, 14.0465}, {2.61529, 20.}}	{16.1736, 47.1806}	inf	121.	393.268	{205.629, 683.302}	129.423	0.322669	newly	167.	191.95	{85., 340.}	41.1916	0.161997	Reff	1.89271	1.95974	{1.52926, 2.59907}	15.7751	ar	97 413.	77 940.	{62 958., 92 089.}	19.9902
40	R0	{2.64218, 18.5237}	{2.07427, 17.1745}	{{1.44118, 12.4645}, {2.8748, 20.}}	{24.5444, 14.5343}	inf	208.	379.398	{185.905, 703.081}	58.0888	0.194943	newly	187.	199.28	{82., 364.}	47.754	0.210387	Reff	2.62693	2.04672	{1.43593, 2.8462}	24.763	ar	99 474.	78 868.1	{57 526., 95 423.}	20.7148
41	R0	{1.6945, 7.17996}	{1.93361, 13.9067}	{{1.20956, 6.16453}, {2.85956, 19.5242}}	{30.9658, 99.7171}	inf	3.	161.279	{8.26673, 358.316}	3137.85	1.32479	newly	9.	115.68	{4., 271.}	1204.44	1.03069	Reff	1.69433	1.88351	{1.15086, 2.7524}	29.9992	ar	96 332.	63 906.7	{27 943., 92 658.}	33.6599
42	R0	{1.84559, 18.0074}	{1.80798, 19.7466}	{{1.70011, 19.8639}, {1.89471, 20.}}	{3.59904, 11.3333}	inf	1046.	1031.93	{851.37, 1210.53}	34.0917	0.184335	newly	870.	419.88	{338., 490.}	51.7379	0.321262	Reff	1.78061	1.78349	{1.67533, 1.86256}	3.77827	ar	97 358.	77 964.	{73 944., 81 427.}	19.9203
43	R0	{2.24965, 17.0361}	{2.01463, 18.059}	{{1.55734, 13.8551}, {2.64266, 20.}}	{19.2306, 15.9355}	inf	172.	406.515	{189.631, 680.613}	74.4286	0.208931	newly	161.	199.82	{77., 335.}	51.2422	0.187207	Reff	2.23721	1.98556	{1.52677, 2.60002}	19.7687	ar	98 770.	78 658.4	{63 695., 92 761.}	20.362
44	R0	{2.54324, 15.7967}	{1.92378, 18.2802}	{{1.53578, 14.9926}, {2.60757, 20.}}	{25.2685, 18.4043}	inf	68.	340.788	{179.395, 593.502}	191.272	0.427908	newly	107.	160.09	{72., 279.}	63.5047	0.204793	Reff	2.53714	1.90586	{1.53009, 2.58517}	25.672	ar	99 354.	76 674.9	{63 110., 92 292.}	22.8266
45	R0	{2.54943, 14.8032}	{2.02387, 16.5264}	{{1.3704, 11.1638}, {2.96479, 20.}}	{25.7947, 23.5722}	inf	40.	236.831	{86.0597, 417.827}	315.821	0.553014	newly	50.	133.89	{46., 224.}	174.26	0.379213	Reff	2.54637	1.99404	{1.36576, 2.93311}	26.2162	ar	99 380.	74 831.1	{49 813., 94 672.}	24.702
46	R0	{2.37755, 12.6461}	{1.93444, 13.9966}	{{1.19018, 6.18069}, {2.85956, 19.5242}}	{25.9247, 33.7981}	inf	8.	161.443	{8.26673, 358.316}	926.879	0.918564	newly	26.	112.05	{4., 261.}	360.115	0.717474	Reff	2.37696	1.8832	{1.15086, 2.7524}	26.6097	ar	99 096.	63 657.6	{19 155., 92 658.}	35.7617
47	R0	{1.69279, 11.6636}	{1.86076, 18.5166}	{{1.58351, 13.6213}, {2.13603, 20.}}	{12.4821, 59.1251}	inf	138.	435.307	{262.247, 592.308}	115.499	0.313411	newly	167.	196.56	{109., 277.}	34.4072	0.130643	Reff	1.68417	1.84069	{1.57913, 2.11878}	11.7879	ar	96 214.	77 301.6	{65 656., 86 092.}	19.6566
48	R0	{1.82402, 10.0038}	{1.99649, 16.0006}	{{1.35208, 9.87798}, {2.75368, 20.}}	{25.1806, 63.8182}	inf	25.	247.743	{84.3628, 415.564}	650.737	0.809346	newly	39.	146.89	{44., 257.}	281.	0.511336	Reff	1.82252	1.9654	{1.31731, 2.74277}	24.7478	ar	97 076.	73 696.3	{45 527., 93 085.}	24.0839
49	R0	{1.6424, 11.1667}	{1.90804, 16.8874}	{{1.46065, 11.7363}, {2.62113, 20.}}	{22.4213, 54.5572}	inf	267.	456.231	{249.59, 759.577}	40.309	0.152846	newly	380.	233.55	{122., 359.}	40.7553	0.261944	Reff	1.62686	1.88498	{1.43265, 2.60315}	22.2202	ar	95 546.	74 489.2	{57 290., 93 055.}	22.0528
50	R0	{2.05638, 14.3048}	{2.08998, 17.2972}	{{1.52633, 13.0282}, {2.87281, 20.}}	{20.7003, 27.3993}	inf	167.	370.803	{189.005, 665.346}	74.9102	0.209738	newly	176.	193.39	{90., 366.}	43.858	0.17794	Reff	2.0461	2.06742	{1.50113, 2.66359}	20.4777	ar	98 194.	79 751.1	{62 627., 94 234.}	18.7821
